# Feasibility of a Hydraulic Power Assist System for Use in Hybrid Neuroprostheses

**DOI:** 10.1155/2015/205104

**Published:** 2015-03-18

**Authors:** Kevin M. Foglyano, Rudi Kobetic, Curtis S. To, Thomas C. Bulea, John R. Schnellenberger, Musa L. Audu, Mark J. Nandor, Roger D. Quinn, Ronald J. Triolo

**Affiliations:** ^1^Advanced Platform Technology Center, Louis Stokes Cleveland Department of Veterans Affairs Medical Center, Cleveland, OH 44106, USA; ^2^Department of Biomedical Engineering, Case Western Reserve University, Cleveland, OH 44106, USA; ^3^Department of Mechanical and Aerospace Engineering, Case Western Reserve University, Cleveland, OH 44106, USA; ^4^Department of Orthopaedics, Case Western Reserve University, Cleveland, OH 44106, USA

## Abstract

Feasibility of using pressurized hydraulic fluid as a source of on-demand assistive power for hybrid neuroprosthesis combining exoskeleton with functional neuromuscular stimulation was explored. Hydraulic systems were selected as an alternative to electric motors for their high torque/mass ratio and ability to be located proximally on the exoskeleton and distribute power distally to assist in moving the joints. The power assist system (PAS) was designed and constructed using off-the-shelf components to test the feasibility of using high pressure fluid from an accumulator to provide assistive torque to an exoskeletal hip joint. The PAS was able to provide 21 Nm of assistive torque at an input pressure of 3171 kPa with a response time of 93 ms resulting in 32° of hip flexion in an able-bodied test. The torque output was independent of initial position of the joint and was linearly related to pressure. Thus, accumulator pressure can be specified to provide assistive torque as needed in exoskeletal devices for walking or stair climbing beyond those possible either volitionally or with electrical stimulation alone.

## 1. Introduction

Restoring walking is one of the main desires of individuals who have a spinal cord injury (SCI) [1]. Thus, there is a significant effort in the field of orthotics to restore locomotion, including walking on uneven terrain and negotiating stairs. There are a number of approaches to restoring upright mobility after paralysis from SCI including mechanical bracing [2], functional neuromuscular stimulation (FNS) [3–5], powered exoskeletons [6–9], and hybrid systems [10, 11] that combine two or more of these approaches.

Recently, there has been a surge in powered exoskeleton development, including commercial devices such as Rewalk and Ekso [12, 13]. Exoskeletons are able to drive the motion of the limbs for walking and stair climbing with external motors. However, they are limited by the amount of power and the weight of the torque generating motors that must be carried. In addition, they do not provide the physiological benefits of exercise from actively contracting the otherwise paralyzed muscles.

Therefore, there has been an increased effort in hybrid system development that combines either surface [14] or implanted FNS [15] with exoskeleton's on-demand external power to provide physiological benefits and to minimize external power requirements. There are a number of ways to provide external power including the use of electric motors [12, 13], pneumatic [16–18] and elastic elements [19–21] to supply joint torques. Other approaches store energy from stimulated knee extensors and transfer it to drive hip motion using springs and pneumatic components [22, 23].

In this study, feasibility of a power assist system (PAS) using a hydraulic circuit with pressurized fluid to provide assistive torque for joint movement was designed and tested. We hypothesized that this PAS could be a viable alternative in providing assistive torque for joint movements.

## 2. Materials and Methods

### 2.1. Power Assist System Design

**Figure 1:**
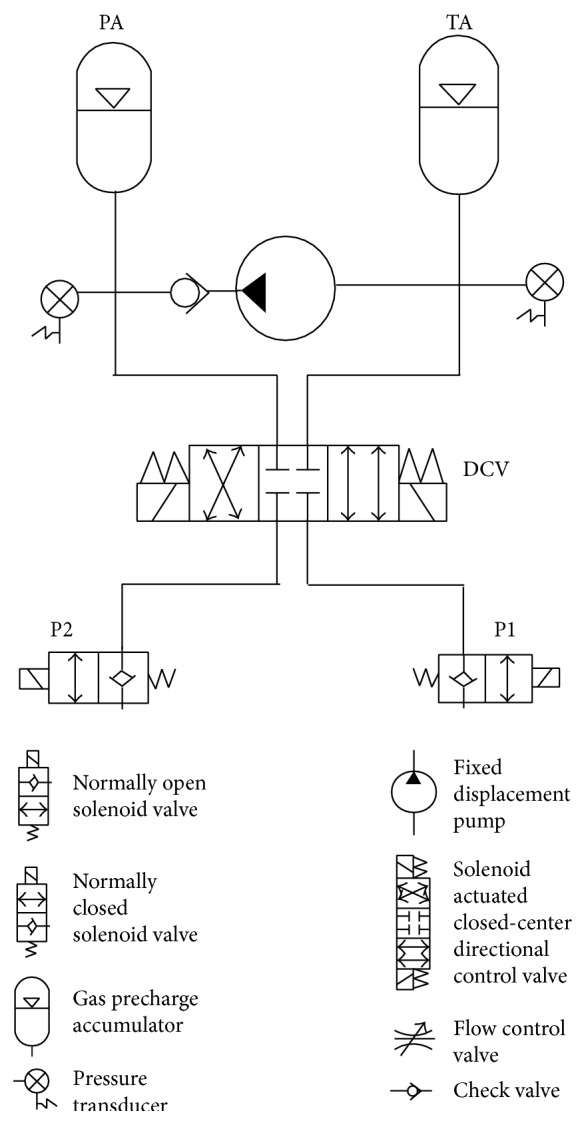
Diagram of the PAS system. Note that P1 and P2 can connect to any hydraulic circuit. In this study, the PAS is connected to a single hydraulic actuator via valve P1.

**Figure 2:**
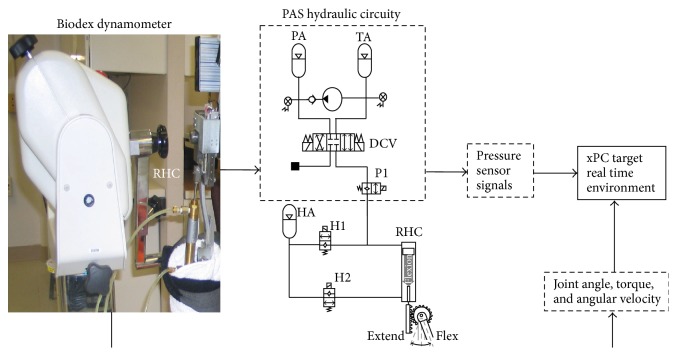
PAS bench testing setup. The PAS hydraulic circuitry is connected to the rotary actuator consisting of right hip cylinder (RHC) connected between the exoskeleton upright and rack and pinion transmission to transmit torque. The upright is attached to a Biodex dynamometer to measure flexion torque. Pressure, torque, angle, and angular velocity data are read into an xPC Simulink real time environment.

As a test bed for bench testing, the PAS (Figure 1) was incorporated into the right hip mechanism of the existing exoskeleton [15] (Figure 2). The PAS utilizes a single pump to pressurize fluid in an accumulator and a directional control valve to distribute it to the hydraulic pistons located at the joints. The previously designed variable constraint hip mechanism [24] utilizes a hydraulic circuit to unlock the swing hip by opening appropriate valves while the stance hip is locked to provide postural support. In this design iteration, linear displacement of the hydraulic cylinder, which was attached to the thigh upright, by the pressurized fluid generates torque at the right hip joint by means of a rack and pinion transmission.

While the PAS system can be applied to any hydraulic circuit, its operation in this design configuration (Figure 2) is as follows. Two valves (H1 and H2) allow the ability to lock, unlock, and isolate the right hip cylinder (RHC) used for torque generation from the PAS. The blind side of the RHC is connected to the pressurized accumulator (PA) by opening the valve P1 and shifting the position of a closed-center 4-way, 3-position solenoid actuated directional control valve (DCV) to the right. Flow from the PA pressurizes the blind side of the RHC, resulting in RHC piston extension which produces a hip flexion torque through the rack and pinion transmission. Fluid from the rod side of the cylinder flows through the open valve H2 into the hip accumulator (HA).

Once the power assist torque is no longer required, the DCV is deenergized shifting the valve left to the closed center position, and the system returns to its default state. To recharge PA to prepare for the next cycle, the pump is activated to transfer fluid from the tank accumulator (TA) to PA. This pressurizes PA to its initial condition (measured by the pump side pressure transducer) and at the same time reduces the pressure of TA below its original system resting pressure. The fluid volume pumped from TA to PA is equal to that displaced from PA to the blind side of the RHC during the hip flexion assist. Next, the DCV is shifted in the opposite direction and valves H1 and P1 are opened to channel the flow into TA from HA. Since TA is below the system resting pressure and the rest of the circuit is above, the excess fluid from the previous PAS injection period stored in HA will then flow back into TA bringing the entire system to its initial condition and ready for the next cycle when needed.

### 2.2. Power Assist System Specifications

To specify individual components of the PAS, biomechanical requirements of stair ascent in normal individuals were examined while considering the limitations of weakened muscles. A minimum of 60° hip flexion angle is necessary for a person of an average stature to clear a standard stair step riser height of 20 cm [26]. Approximately 23 Nm of hip flexion moment is necessary to hold a leg at 60° hip flexion for a 79 kg individual [26]. Therefore, we specified PAS components to generate 25 Nm of torque at 60° of hip flexion. The volume (*V*_PA_) of the hydropneumatic accumulator, PA, derived from Boyle's law, can be expressed as in (1) (note that the influences of temperature and system efficiency were not factored into the derivation of (1) and the compressible fluid in the accumulator is nitrogen). Consider the following:

(1) $$\begin{array}{c} V_{\text{PA}}=\frac{A_{\text{blind}}r\Delta \theta_{\text{hip}}}{0.9^{1/n}-{\left(0.9\left(\tau_{\text{end}}+p_{\text{rest}}A_{\text{rod}}r\right)/p_{\text{ic}}A_{\text{blind}}r\right)}^{1/n}}, \end{array}$$

where *A*_blind_ is the RHC piston blind area, *A*_rod_ is the RHC piston rod area, *r* is linear-to-rotary rack and pinion transmission ratio (0.55 mm/deg [15]), Δ*θ*_hip_ is the estimated hip angle excursion that may require power assist, *n* is the polytropic index (1.4 for nitrogen assuming an adiabatic process), *τ*_end_ is the output torque at the end of the hip motion (25 Nm), *p*_rest_ is the system resting pressure (414 kPa), and *p*_ic_ is the initial pressure of PA and corresponds to the operating pressure rating of the pump. Thus, the objective is to minimize *p*_ic_ so that the power requirements of the pump are minimized. Equation (1) can be solved for *p*_ic_, to get

(2) $$\begin{array}{c} p_{\text{ic}}=\frac{0.9\left(\tau_{\text{end}}+p_{\text{rest}}A_{\text{rod}}r\right)}{A_{\text{blind}}r{\left(0.9^{1/n}-A_{\text{blind}}r\Delta \theta_{\text{hip}}/V_{\text{PA}}\right)}^{n}}. \end{array}$$

All the variables on the right side of (2) are known except for *V*_PA_ and Δ*θ*_hip_. Knowing this and assuming the values are as stated above, it can be seen upon inspection of (2) that *p*_ic_ decreases with an increasing *V*_PA_ and decreases with a decreasing Δ*θ*_hip_. Thus, the goal is to minimize the required Δ*θ*_hip_ and choose a *V*_PA_ such that it is small enough to be portable but large enough so that *p*_ic_ is kept low. For example, if we assume that power assist is necessary from 0 to 60° hip flexion and choose a small diaphragm accumulator of 0.075 L (Hydac, Bethlehem, PA) for PA, *p*_ic_ would need to be approximately 3213 kPa. In this case, when the DCV and P1 are opened to connect PA to the cylinder at 0° hip flexion, 3213 kPa of fluid pressure at the cylinder produces 35 Nm of hip flexion torque by means of the linear-to-rotary transmission. The pressure drops to 2413 kPa at 60° hip flexion to produce 25 Nm.

Another critical specification of the pump is the displacement or flow output. A high displacement allows for a faster recharge time of PA. In this example, if we assume that it is necessary to recharge PA within one second, the pump displacement needed is 12.9 cm^3^/s at 2413 kPa. However, if we assume power assist is only necessary from 30 to 60° hip flexion and choose a larger accumulator of 0.16 L, *p*_ic_ would drop to 2565 kPa. In this case, at the onset of power assist, 2565 kPa at the cylinder produces 27 Nm of hip flexion torque at 30° hip flexion. The pressure then decreases to 2413 kPa at 60° hip flexion to produce 25 Nm. Since Δ*θ*_hip_ is reduced to half of that in the previous example, the required pump displacement is also reduced by half to 6.45 cm^3^/s at 2413 kPa.

A major advantage of a hydraulic power source is the potential weight reduction. Hydraulic systems have been shown to have torque/mass ratios many times greater than that of electric motors and other power sources [27]. In addition, the system described here contains just one motor/pump unit that can be placed proximally on the exoskeleton and direct power distally to the joints as needed as opposed to having a motor at each joint. By locating the pump proximally the need for additional assistive power due to the inertial demands of distally located weight is minimized. Keeping the mass proximal allows it to be directly transferred to the ground and is not carried by the user or an external power source [25]. Suitable wearable miniature hydraulic pumps are currently being developed [28] and adding this unit to our current exoskeleton prototype will keep the weight (Table 1) at or below clinically available electrically driven exoskeletons [29].

### 2.3. Power Assist System Simulation

**Figure 3:**
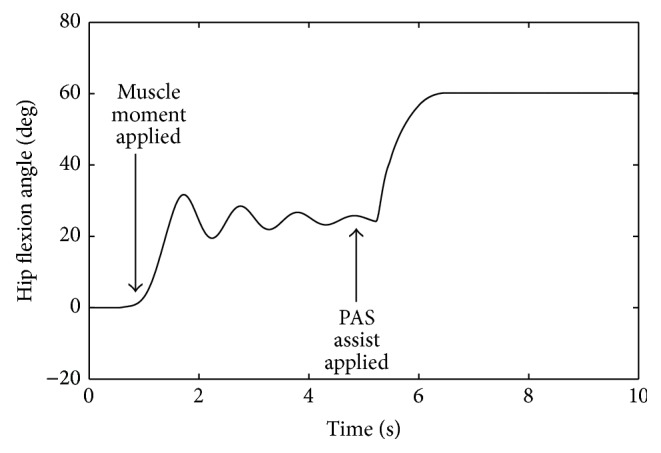
Simulated PAS operation. Hip angle of 25° was achieved before activation and assistive power of PAS was simulated to drive hip to 60° of flexion.

A computer model was created in MATLAB (The MathWorks, Inc., Natick, MA, USA) using SimMechanics, SimHydraulics, and SimScape Toolboxes of Simulink to verify the proposed PAS design. In the simulation, the PAS was connected to an anatomically realistic model of the human leg which simulated hip flexion moment on movement produced through either volitional contraction or FNS. A hip flexion torque of 20 Nm was applied to the leg model 1 second after initiating the simulation which resulted in a hip rotation of 25°. The simulated hydraulic valves of the PAS model were then opened to expose the cylinder to the high pressure accumulator to apply an external assistive hip flexion torque, which resulted in a further increase in hip flexion to the specified angle of 60° (Figure 3).

### 2.4. Power Assist System Bench Testing

A prototype of the hydraulic PAS was constructed from off-the-shelf components for bench testing. For simplicity, only the right hip was included for bench testing as shown schematically in Figure 2. The directional control valve (DCV) (Hyvair Corporation, Magnolia, TX) and solenoid valves P1 and H1 (Allenair Corporation, Mineola, NY) controlled fluid flow between pressurized accumulator PA and the 2.2 cm bore double-acting hydraulic cylinder RHC (Clippard Minimatic, Cincinnati, OH). The power of pressurized fluid stored in PA, a 0.075 L diaphragm style accumulator (Hydac, Bethlehem, PA), was released to produce force on the cylinder's piston which provided torque at the hip by means of a rack and pinion transmission.

Torque, angle, and angular velocity measurements were made with a Biodex System 3 robotic dynamometer (Biodex Medical Systems, Shirley, NY). Pressure sensors (Gems Sensors & Controls, Plainville, CT) were attached to monitor pressure of accumulators PA and TA at the blind and rod side of the cylinder. Data were collected with custom circuitry integrated into the MATLAB xPC target prototyping environment which executes Simulink models in real time. A NI 6071E DAQ card (National Instruments, Austin, TX) inside of the target PC acquired torque, angle, and angular velocity from the Biodex. In addition, the DAQ card delivered digital output signals from the xPC target to control the valves.

The hydraulic cylinder attached to the thigh upright provided hip flexion by linear displacement of the piston through the rack and pinion transmission. The thigh upright of the brace was attached to the Biodex arm (Figure 2). The entire hydraulic system with all valves in the open position was precharged to 414 kPa to minimize the compliance from any residual air bubbles left in the system. The high pressure accumulator (PA) was pressurized with a hand pump with all valves in their default states as seen in Figure 2. Once the desired pressure was reached, the DCV and appropriate hydraulic valves were opened to expose the right hip cylinder to the high pressure which applied torque at the hip. The DCV was shifted to the right and P1 was opened for fluid flow from the PA to the blind side of the RHC to extend the cylinder which flexed the hip. The excess fluid from the rod side of the cylinder went into the accumulator HA. In a portable design a motorized pump would move fluid from TA to PA. Once the flexion stage was complete, the DCV was shifted to the left while the P1 stayed open and H2 closed to shift the excess fluid back into TA. The PA was pressurized to 689, 1379, 2068, 2758, and 3172 kPa (maximum safe rating for components used) for isometric tests at 0 and 60° of hip flexion. In addition, isokinetic tests limited to 120°/s were performed to measure the differential pressure in the cylinder to calculate the torque throughout the entire range of motion.

To determine passive resistance of the hydraulic circuit of the PAS, the hip was flexed through the range of motion by pulling vertically on a load cell through a rope which was attached to the brace upright for six trials. The force measured by the load cell was multiplied by the perpendicular distance from the line of force application to the hip center to calculate the moment required to flex the hip. This distance was found by attaching reflective markers at the hip center, the point of force application on the upright and on the rope using a Vicon (Oxford, UK) motion capture system to track the movement and determine the changing moment arm. This was repeated for six trials with the hydraulic cylinder detached from the rack and pinion transmission to find the difference in moment caused by the passive resistance of the hydraulic circuit.

### 2.5. Power Assist System Human Testing

To simulate PAS operation with an SCI subject, an able-bodied volunteer (165 cm, 64 kg) gave her informed consent and was fitted with the brace. Reflective markers were placed on the trunk corset and leg upright and the Vicon motion capture system was used to capture the marker trajectories at 120 Hz and body segments were created from the marker data to calculate joint angles. The high pressure accumulator was charged to 3172 kPa. To simulate voluntary muscle weakness or the actions of FNS, the subject was instructed to flex her right hip to approximately 45° after which the PAS was activated to complete the movement while she was instructed to provide minimal resistance or assistance. Six trials with PAS were performed and the data were averaged across the six trials.

## 3. Results

### 3.1. Power Assist System Bench Testing

**Figure 4:**
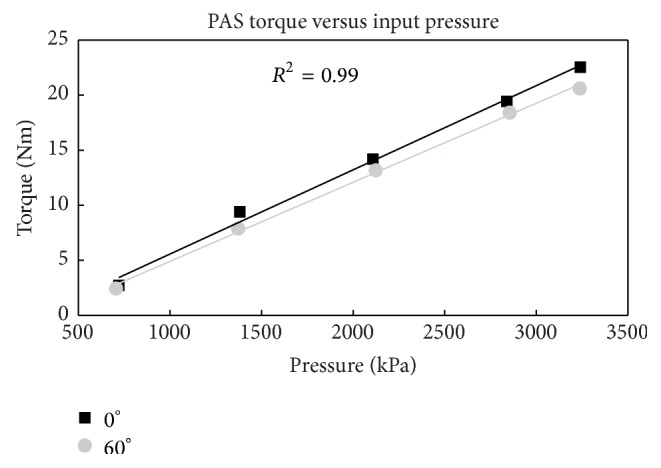
Output isometric torque as a function of supplied pressure at 0 and 60° of hip flexion. *R* ^2^ of 0.99 is representative of both curves.

**Figure 5:**
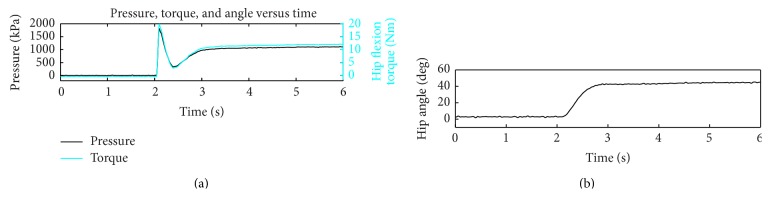
Cylinder pressure differential and torque change (a) during isokinetic (120°/s) movement of the hip (b). Power assist was activated at time = 2 seconds.

The input pressure supplied to the PA accumulator and the output isometric hip flexion torque at 0 and 60° is shown in Figure 4. There is a linear relationship between the input pressure and the output torque and the output torque is practically independent of hip flexion angle. Therefore, we can control the applied hip flexion torque by controlling the pressure of the accumulator.  Figure 5 shows a typical change in hip angle, torque, and cylinder pressure differential in the isokinetic tests. When a pressure of 3172 kPa was released with the orthosis in full hip extension, the resulting torque of about 20 Nm moved the hip into 50° of hip flexion. The torque decreased as the hip moved into flexion due to the decrease in pressure with extension of the hydraulic cylinder flexing the hip.

The mean angular velocity of the hip was 77 ± 8°/s with a mean flow rate of 16.5 ± 1.6 cm^3^/s. The average system response time was 93 ± 23 ms from the activation of the DCV to onset of hip movement. Passive resistance of the PAS circuit was found to be less than 4 Nm throughout the range of motion.

### 3.2. Power Assist System Human Testing

**Figure 6:**
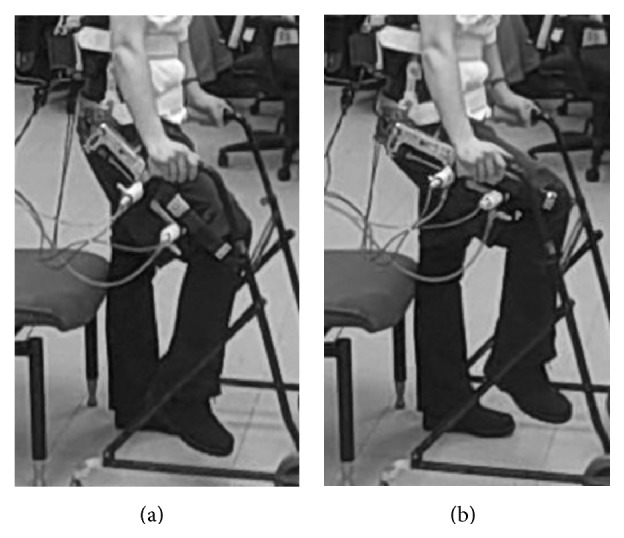
Able-bodied PAS test. Subject simulated FNS by flexing hip to approximately 45° (a). PAS was then activated to continue flexing the hip to approximately 77° with minimal user assistance or resistance (b).

In the able-bodied experiments, the PAS increased hip flexion angle by a mean of 32 ± 5° across the six trials. Photographs before and after the PAS was activated are shown in Figure 6 to illustrate the additional hip flexion that was provided following volitional flexion.

## 4. Discussion and Conclusions

A hydraulic source of assistive power was designed and tested in this study. The PAS was able to provide 21 Nm of torque to supplement hip flexion in a hybrid system combining weak voluntary muscle activity and an exoskeleton. More importantly, our results validate a linear relationship between input pressure and output torque that is independent of joint angle. For preliminary assessment, the accumulator was pressurized using a simple hand pump; however, automated pumps and motors capable of producing required pressures with reasonable weight and power consumption for portable use are available [28] and can be integrated into the PAS in the future now that feasibility of the approach has been established.

The torque values measured during bench testing were lower than the values calculated from the mathematical equations and model (21 Nm as compared to the predicted 25 Nm at 60°). This difference is likely due to the passive resistance caused by friction in the hydraulic system and the linear-to-rotary transmission. The pressure drop across the cylinder was higher than predicted from the model (ending pressure of approximately 1379 kPa as compared to the predicted 2413 kPa). This is likely due to residual air in the system and suboptimal component selection. Custom fittings or a manifold designed specifically for the circuit has the potential to reduce the number of hydraulic connectors and the amount of tubing which could minimize the pressure losses and reduce passive resistance.

We were able to achieve 32° of assistive hip flexion in the able-bodied testing. While it would have been beneficial to have electromyogram (EMG) recordings of the subject to ensure there was no volitional assist once the PAS was activated, the major muscles responsible for hip flexion are deep within the pelvis and not easily accessible for EMG recording. Thus, the able-bodied testing was only to approximate the results for an SCI subject using FNS and continued clinical testing with volunteers with paraplegia is necessary.

From the results of the bench testing, we can extrapolate and predict pressures that would be required to produce a desired hip flexion moment. Knowing the relationship between input pressure and torque and the pressure drop across the range of motion, the pressure required to achieve desired hip flexion motion can be specified. For example, if it takes 23 Nm of hip flexion moment at 60° for a leg to clear a standard step [26] plus additional 15 Nm for the 5 kg knee-ankle-foot orthosis [15], then an input pressure of approximately 6440 kPa would be required to flex the hip from 0° to 60° assuming the worst case scenario of no volitional or stimulated input from the user. This includes the pressure of 5240 kPa required to generate 38 Nm extrapolated from Figure 4, in addition to compensating for the observed pressure drop of 20 kPa/° as the hip flexes (Figure 5). However, if a stimulated response can achieve 25 Nm at 60° [26], the remaining torque could be added by using an input pressure of approximately 3447 kPa to supplement the stimulation. All of these pressures are achievable and safe with proper component selection; however, this feasibility testing was limited to the existing setup and components and was constrained to a maximum of 3172 kPa due to the limitations of the accumulators and tubing of our current hydraulic system.

Preliminary results show that using readily available off-the-shelf hydraulic components it is feasible to integrate a power assist system into a hybrid neuroprosthesis in order to provide additional hip flexion torque to assist in lifting and supporting the braced leg to a desired height. Controllers can be developed to combine power assist on demand with muscle stimulation [30] to maximize physiological benefits of stimulation and minimize external power requirements.

An advantage of this hydraulic design is that, with proper valve configuration [24], it is possible to transmit hip flexion power from one hip to another to assist with contralateral extension and to the knee through hip-knee coupling without requiring a dedicated motor at each joint. Thus, a similar system that contains just one motor/pump unit can be constructed to direct power to whichever joint it needed as opposed to using a motor at each joint to be actuated.

There are three major advantages of using flow discharged from an accumulator as opposed to using flow directly generated from a pump. First, pressure does not have to build from a low pressure which would result in a slow application of torque. High pressure is applied to the cylinder immediately after the DCV has opened which will enable a response time comparable to stimulated muscle. While the average system response time from valve activation to limb movement was 93 ms, isometric testing found that the time from valve activation to 90% of the maximum torque output was 159 ± 45 ms, which falls within range of stimulated muscle response times (100–300 ms) [31]. This response time was independent of input pressure but likely will be influenced by component selection (e.g., tubing and valve switching speed) and any compliance or residual air bubbles in the system. Due to this quick response time, a small fixed displacement pump can be used rather than a larger and costlier pressure-compensated variable displacement pump. The second advantage of using an accumulator to discharge the flow is that pump activation can be kept separate from valve activation. This reduces the maximum instantaneous electric power requirements to drive the hydraulic system. And third one is for safety in that the hydraulic exoskeleton worn by the user is never directly coupled to the pump which will reduce the risk of a malfunctioning pump injecting too much pressure which could harm the individual by moving the joints to undesired positions.

Since the PAS operation is feasible, future studies need to concentrate on quantifying system performance in volunteers with SCI in conjunction with FNS. The PAS approach can also be applied without stimulation to assist physiological or household ambulators with partial paralysis or significant muscle weakness and limited voluntary movement. Future technical development should include system optimization to minimize the weight and power consumption of the system and maximize the pressure and displacement that the pump supplies to accumulator.

## Figures and Tables

**Table 1:** Weight of HNP + PAS^*^.

Structure	6.5 kg
Hydraulics	7.4 kg
Battery/electronics	1.5 kg
Pump^*^ [28]	2.75 kg

Total	18.15 kg

Weight of entire hybrid neuroprosthesis with ^*^pump for power assist system added.

## Abbreviations

DCV: Directional control valve
EMG: Electromyogram
FNS: Functional neuromuscular stimulation
HNP: Hybrid neuroprosthesis
PA: Pressurized accumulator
PAS: Power assist system
RHC: Right hip cylinder
SCI: Spinal cord injury
TA: Tank accumulator.
